# Editorial: Advances in flow-diversion devices for cerebral aneurysms

**DOI:** 10.3389/fneur.2023.1195367

**Published:** 2023-07-04

**Authors:** Xianli Lv

**Affiliations:** Department of Neurosurgery, School of Clinical Medicine, Beijing Tsinghua Changgung Hospital, Tsinghua University, Beijing, China

**Keywords:** flow-diversion, cerebral, aneurysm, endovascular, treatment

I am honored to co-edit the topic “*Advances in flow-diversion devices for cerebral aneurysms*” with Pervinder Bhogal and Alexander Sirakov. The material of this Research Topic has published cutting-edge research on this subject from all over the world, including clinical application research of new flow-diversion devices (FDs), FD treatment of distal aneurysms, ruptured and unruptured dissecting aneurysms, bifurcation aneurysm, antiplatelet therapy and hemodynamic study of FDs.

In the past 60 years, great success has been achieved in endovascular surgery of cerebral aneurysms. From the iron-acrylic compound (1), electrocoagulation, detachable balloon in the 1960s to the electrolytic coil in 1990s, the endovascular surgery became an alternative to surgical clipping for cerebral aneurysm treatment. The FD emerged in 2007 has fundamentally changed the previous concept of endovascular surgery of cerebral aneurysms, from intra-aneurysm filling, occlusion of the parent artery, clipping of the aneurysm neck, remodeling of the aneurysm orifice to repair the pathological arterial wall (2). This is undoubtedly correct, because the basic pathological changes for the occurrence of an aneurysm are the weakness of the intima, atherosclerosis, injury, or infection of the arterial wall. FD has greatly expanded the scope and improved the outcome of endovascular surgery of cerebral aneurysms, from large and giant to tiny aneurysms, from unrupture to ruptured blister-like/dissecting aneurysms, from side-wall to bifurcation aneurysms (3). FDs make those complex aneurysms, which are incurable by previous endovascular techniques, to be curable.

The concept of FD is derived from the experiences and lessons learned in the development of stent-assisted coiling, which provides the denser coil filling, better angiographic and clinical results. Stent-assisted coiling directly interrupts the blood flow from the parent artery into the aneurysm and leads to the thrombosis in the aneurysm sac. The concept FD is based on two principles: (1) placing woven mesh device in the parent artery changes the blood flow away from the aneurysm sac and (2) the device structure provides a scaffold for the growth of endothelial cells. This process of “new endothelialization” separates the aneurysm from the circulation and allows the gradual thrombosis of the aneurysm (4). The advantage of FD is that it can repair the weakened arterial wall with a very low recurrence rate contrasting to the high recurrence rate associated with coil embolization. In addition, when deploying the FD, the devices does not need to enter the aneurysm sac directly, thus lowered the risk of aneurysm rupture during treatment.

As the first FDA certified FD, the pipeline embolization device (PED; Medtronic Neurovascular, Irvine, California, USA) is still the most popular FD at present. Its safety and effectiveness of the off-label use have been widely published. Its clinical use has expanded from unruptured internal carotid artery aneurysms to the most cerebral aneurysms, such as anterior cerebral artery, middle cerebral artery and posterior circulation aneurysms (including posterior cerebral artery and posterior inferior cerebellar artery aneurysms; Figures 1A–D). It is a good option for ruptured and unruptured aneurysms with incorporated perforating arteries. FD has become the first-line treatment for blister-like aneurysms (5). There are also reports on the FD treatment of carotid cavernous fistulas. In our center, the FD has replaced the coil embolization in most of unruptured aneurysms due to its low complication rate, low recurrence rate and high occlusion rate (3). In a meta-analysis including 11 studies (6), the occlusion rates of unruptured aneurysms at 1 year, 1–2 years, 2 years, 3 years, and 5 years follow-up were 77%, 87.4%, 84.5%, 89.4%, and 96%, respectively. There were 5% long-term in-stent stenosis, one delayed ischemic stroke and no delayed hemorrhage of aneurysm.

**Figure 1:**
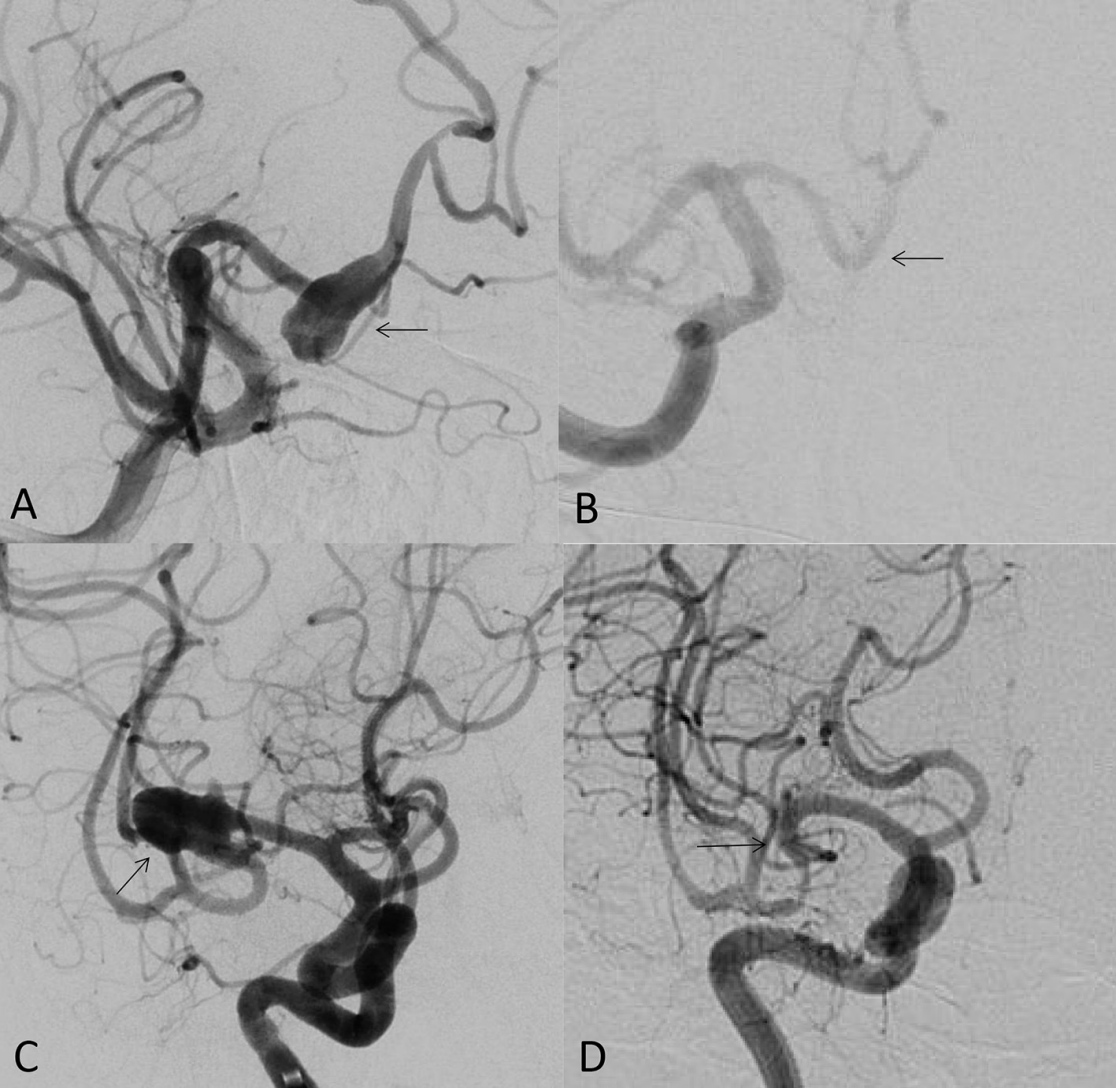
**(A)** 49-year-old male, unruptured aneurysm of the right anterior cerebral artery (arrow); **(B)** the 7 months control angiogram after PED treatment showed that the aneurysm was completely occluded (arrow); **(C)** 53-year-old female, the right middle cerebral artery bifurcation aneurysm (arrow); **(D)** the control angiogram after 9 months showed that the aneurysm was completely occluded (arrow).

There are many other FDs with different designs (2), such as Silk, Silk+ and Silk vista baby (Balt Extrusion; Montmorency, France); Surpass Streamline and Surpass Evolve (Stryker Neurovascular, Fremont, CA); The FRED and FRED Jr (Microvention, Aliso Viejo, California); p64, p48MW, and p48-HPC (Phenox, Bochum, Germany); Derivo Embolization Device (Acandis GmbH, Pforzheim, Germany); Tubridge (MicroPort, Shanghai, China) and Lattice (Accumedical, Beijing, China). Up to date, the FDA approved PED in 2011, Surpass in 2018 and FRED in 2019, all of which are used to treat large or large wide-necked intracranial aneurysms along the internal carotid artery (FDA.gov). All FDA-approved FDs consist of Nitinol or Co-Cr alloy. If the clinical scenario requires, the endovascular neurosurgeon must be proficient in at least one or two of them as well as maintain a certain understanding and familiarity with the advantages/limitations of these devices.

The main limitation of FD is ischemic stroke associated with intra-stent thrombosis, which requires dual antiplatelet therapy or GP IIb/IIIa inhibitors (7). Therefore, we must bear their related bleeding risks. Different manufacturers have designed antithrombotic coating FDs to reduce the risk of thrombosis since 2014. At present, the available antithrombotic coating FDs include Pipeline Flex embolic device with shielding technology (Medtronic Neurovascular, Irvine, California, USA), Derivo embolic device (Acandis GmbH, Pforzheim, Germany) and p48-HPC (Phenox, Bochum, Germany). Preliminary follow-up data shows that the incidence of ischemia related to FDs is low and their angiographic occlusion rate is comparable to uncoated FDs.

With the increase in the off-label use of FDs in a broader clinical scenario (8), it is necessary to design and customize the specific aneurysm anatomical structure, location and shape to cover a broader range of clinical indications. The use of FDs in small arteries has been widely reported (9). The most commonly used device is PED, as well as FRED and SILK series. Few studies support the efficacy of Surpass, p64, Derivo, and Tubridge FDs in small arteries.

Finally, we thank all the authors and endovascular centers who have contributed to this topic and congratulate their great success in the use of FDs. This topic will undoubtedly continue to arouse the enthusiasm of the research, development and clinical use of FDs, and pursuit of excellent endovascular surgery results of cerebral aneurysms.

## Author contributions

The author confirms being the sole contributor of this work and has approved it for publication.
